# Mining the phytomicrobiome to understand how bacterial coinoculations enhance plant growth

**DOI:** 10.3389/fpls.2015.00784

**Published:** 2015-09-24

**Authors:** Maskit Maymon, Pilar Martínez-Hidalgo, Stephen S. Tran, Tyler Ice, Karena Craemer, Teni Anbarchian, Tiffany Sung, Lin H. Hwang, Minxia Chou, Nancy A. Fujishige, William Villella, Jérôme Ventosa, Johannes Sikorski, Erin R. Sanders, Kym F. Faull, Ann M. Hirsch

**Affiliations:** ^1^Departments of Molecular, Cell, and Developmental Biology, University of California, Los AngelesLos Angeles, CA, USA; ^2^Bioinformatics, University of California, Los AngelesLos Angeles, CA, USA; ^3^Pasarow Mass Spectrometry Laboratory, Department of Psychiatry and Biobehavioral Sciences, David Geffen School of Medicine at UCLA, Semel Institute for Neuroscience and Human Behavior, University of California, Los AngelesLos Angeles, CA, USA; ^4^Department of Microbiology, Immunology, and Molecular Genetics, University of California, Los AngelesLos Angeles, CA, USA; ^5^Biotechnology, Plants, and Microorganisms Biology, University of Montpellier IIMontpellier, France; ^6^Leibniz-Institut DSMZ-Deutsche Sammlung von Mikroorganismen und Zellkulturen GmbHBraunschweig, Germany; ^7^Molecular Biology Institute, University of California, Los AngelesLos Angeles, CA, USA

**Keywords:** coinoculations, *Bacillus simplex*, genome studies, rhizosphere, legumes

## Abstract

In previous work, we showed that coinoculating *Rhizobium leguminosarum* bv. *viciae* 128C53 and *Bacillus simplex* 30N-5 onto *Pisum sativum* L. roots resulted in better nodulation and increased plant growth. We now expand this research to include another alpha-rhizobial species as well as a beta-rhizobium, *Burkholderia tuberum* STM678. We first determined whether the rhizobia were compatible with *B. simplex* 30N-5 by cross-streaking experiments, and then *Medicago truncatula* and *Melilotus alba* were coinoculated with *B. simplex* 30N-5 and *Sinorhizobium* (*Ensifer*) *meliloti* to determine the effects on plant growth. Similarly, *B. simplex* 30N-5 and *Bu. tuberum* STM678 were coinoculated onto *Macroptilium atropurpureum*. The exact mechanisms whereby coinoculation results in increased plant growth are incompletely understood, but the synthesis of phytohormones and siderophores, the improved solubilization of inorganic nutrients, and the production of antimicrobial compounds are likely possibilities. Because *B. simplex* 30N-5 is not widely recognized as a Plant Growth Promoting Bacterial (PGPB) species, after sequencing its genome, we searched for genes proposed to promote plant growth, and then compared these sequences with those from several well studied PGPB species. In addition to genes involved in phytohormone synthesis, we detected genes important for the production of volatiles, polyamines, and antimicrobial peptides as well as genes for such plant growth-promoting traits as phosphate solubilization and siderophore production. Experimental evidence is presented to show that some of these traits, such as polyamine synthesis, are functional in *B. simplex* 30N-5, whereas others, e.g., auxin production, are not.

## Introduction

Rhizosphere bacteria function as a consortium, synergistically protecting plants from disease (Kloepper et al., 2004), providing plants with essential nutrients (Pradhan and Sukla, 2005; Martínez-Hidalgo et al., 2014), and stimulating plant growth by producing growth-promoting factors (El-Tarabily et al., 2008; Merzaeva and Shirokikh, 2010). Rhizosphere bacteria are analogous to gut bacteria in mammals, which perform similar functions, and like gut bacteria, the microbes that live on and within plant tissues are indispensable for plant survival. Although the microbial composition of the root microbiomes for many plants is known (Schlaeppi et al., 2014), defining the mechanisms driving the microbe/plant synergism in the soil is challenging. This is because soil is complex and the experiments are difficult to perform. Thus, simpler models have been employed, such as using microcosms or rhizotrons and also limiting the number of plant and microbial species to be studied. This is especially true for specific interactions such as those involved in nitrogen fixation, where investigations of the interactions between nitrogen-fixing bacteria and other soil bacteria or fungi consist of coinoculating a legume plant with a rhizobium and a single plant growth promoting bacterial (PGPB) species. Such interactions usually result in an enhancement of plant growth over inoculation solely with rhizobia (see references in Schwartz et al., 2013).

The most frequent bacterial partners in coinoculation studies involving rhizobia are *Bacillus* species, including among others, *B. subtilis, B. amyloliquefaciens, B. licheniformis*, and *B. pumilus*. Earlier, we showed that coinoculating *Pisum sativum* L. with *Rhizobium leguminosarum* bv. *viciae* 128C53 and *B. simplex* 30N-5 resulted in better nodulation and an overall increase in plant dry weight (Schwartz et al., 2013). *B. simplex* 30N-5 is a relatively new player in the panoply of bacteria that positively influence plant growth. This species is mainly known for its phenotypic adaptations with respect to growing on the sun compared to shade walls of “Evolution Canyon” in Israel (Koeppel et al., 2008). However, a number of publications, including our own, have reported that *B. simplex* also functions as a PGPB species (Ertruk et al., 2010; Hassen and Labuschagne, 2010). Recently, the sequenced genomes of several *B. simplex* strains became available and allowed prediction of possible molecular mechanisms for the observed interactions. The essential extension of such genome comparisons include the identification of the expressed proteins, and perhaps most importantly, the identification of the small molecule products of their activity.

In this study, we coinoculated *B. simplex* 30N-5 with either *Sinorhizobium* (*Ensifer*) *meliloti* 1021 (alpha-rhizobium) or *Burkholderia tuberum* STM678 (beta-rhizobium), on their respective hosts. To our knowledge, *Bu. tuberum* STM678 (Moulin et al., 2001; Vandamme et al., 2002) has not been previously employed in coinoculation studies. To obtain a better understanding of the traits that are important for the plant responses in the coinoculation experiments, we analyzed the *B. simplex* 30N-5 genome for genes known to encode PGPB traits. To do this, we compared *B. simplex* 30N-5 with the well-established PGP *Bacillus* strains, namely *B. subtilis* GB03, *B. amyloliquefaciens* subsp. *plantarum* FZB42, and others. In this report, we also demonstrate that several of these PGPB traits are functional in *B. simplex* 30N-5.

## Materials and methods

### Phylogenetic analysis

Nucleotide sequences were obtained from the Joint Genome Institute (IMG/ER) database for microbial genomes (Markowitz et al., 2012). Five housekeeping genes *atpD, urvA, rpoB, lepA*, and *recA* were used to construct concatenated sequences (Table S1). The concatenated gene sequences were aligned with Clustal X (Thompson, 1997), and phylogenetic distances were calculated according to the Kimura two-parameter model (Kimura, 1980). The phylogenetic tree topology was inferred from the maximum-likelihood method employing MEGA5 (Tamura et al., 2011). Confidence levels on each node are the product of 1000 bootstrap replicates.

### Growth of bacteria

*Bacillus* strains were grown on LB (Luria-Bertani; Miller, 1972), Tryptic Soy Agar (TSA; Difco®, Becton Dickenson) or Tryptone Yeast Extract (TY; Beringer, 1974) medium at 30°C or 37°C. Rhizobial strains were cultured at 30°C on either Yeast Mannitol Agar (YMA; Somasegaran and Hoben, 1994) or on TY medium with or without 10 μg/mL tetracycline. *Bu. tuberum* STM678 was grown on LB minus salt or on BSE medium (Caballero-Mellado et al., 2007) with or without antibiotics. Cell density was determined from the OD_600_ nm of the cultures. The bacterial strains studied in this report are listed in Table 1.

**Table 1:** **Strains and plasmids used in this study**.

**Strain number**	**Species name and relevant characteristics**	**Source or reference**
30N-5	*Bacillus simplex*	Schwartz et al., 2013
237	*Bacillus simplex*	Kaplan et al., 2013
11	*Bacillus simplex*	Kaplan et al., 2013
FZB42	*Bacillus amyloliquefaciens* subsp*. plantarum*	*Bacillus* Stock Center
DSM13 Goettingen/ATCC 14580	*Bacillus licheniformis*	*Bacillus* Stock Center
GB03	*Bacillus subtilis*	*Bacillus* Stock Center
NRRL B-4317	*Paenibacillus polymyxa*	*Bacillus* Stock Center
60b4	*Bacillus subtilis*	Flora Pule-Meulenberg
26a1	*Bacillus cereus*	Flora Pule-Meulenberg
HB101	*E. coli*	Cathy C. Webb
Rm1021	Wild-type *Sinorhizobium meliloti*	Lab strain
Rm1021/pHC60	GFP+, Tet^r^ derivative of wild-type *S. meliloti*	This study
STM678	Wild-type *Burkholderia tuberum*	Moulin et al., 2001; Vandamme et al., 2002
STM678/TnGFP	Tet^r^ derivative of wild-type *Bu. tuberum*	Elliott et al., 2007
**Plasmids**	**Relevant characteristics**	**Source or Reference**
pHC60	GFP plasmid, Tet^r^	Cheng and Walker, 1998

To introduce fluorescent markers into *Sinorhizobium* (*Ensifer*) *meliloti* 1021, the plasmid pHC60 (Cheng and Walker, 1998) carrying a green fluorescent protein (GFP) construct was mobilized into *S. meliloti* using a triparental mating procedure (Figurski and Helinski, 1979) as adapted by Schwartz et al. (2013). The *Bu. tuberum* STM678 GFP+ strain was a gift from Dr. J. Peter Young (University of York).

The Voges-Proskauer test (Voges and Proskauer, 1898) was performed as modified by Werkman (1930) and Barritt (1936). Each strain was tested three times.

### Chemical analysis

Cell pellets (1.8 × 10^9^ cells/sample) from *B. simplex* 30N-5 were lysed in 5 ml of either methanol or aqueous trifluoroacetic acid (TFA, 10%) or aqueous trichloroacetic acid (TCA, 8.3%). The homogenates were centrifuged (16,000 × g, 5 min, room temperature) and the supernatants were taken to dryness in a vacuum centrifuge. The dried residue was resuspended in water (500 μL), centrifuged (16,000 × g, 5 min, room temperature) and the supernatant transferred to LC injector vials. For the polyamines, aliquots of the supernatant were injected (8 μl) onto a reverse phase HPLC column (Phenomenex Kinetex C18 100 × 2.1 mm, 1.7 μ particle size and 100 Å) equilibrated in 80% solvent A (0.1 mM perfluoro-octanoic acid in water) and 20% solvent B (0.1 mM perfluoro-octanoic acid in methanol), and eluted (100 μL/min) with an increasing concentration of solvent B (min/%B; 0/20, 5/20, 15/75, 20/75, 22/20, 30/20). The effluent from the column was directed to an electrospray ion source connected to a triple quadrupole mass spectrometer (Agilent 6460) operating in the positive ion tandem mass spectrometric (MS/MS) mode, and the time-dependent intensity of multiple reaction monitoring (MRM) transitions were recorded at previously optimized settings [spermine, m/z (MH^+^) 203
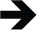
129, 112, 84, fragmentor 55, collision energy 16; spermidine, m/z (MH^+^) 146
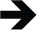
129, 112, and 72, fragmentor 55, collision energy 12; putrescine, m/z (MH^+^) 89
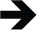
72, fragmentor 40, collision energy 4]. Peak areas for each compound at the corresponding retention times (spermine, spermidine, and putrescine at 16.4, 16.0, and 15.6 min, respectively) were computed with instrument manufacturer-supplied software (Agilent MassHunter). A standard curve was prepared with each experiment from samples containing known concentrations of all three compounds using the signals for the most intense MRM transitions (203
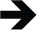
112, 146
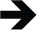
112, and 89
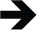
72 for spermine, spermidine, and putrescine, respectively), and the amount of each amine in each biological sample was calculated by interpolation from the standard curves. Under the prescribed conditions, the limit of detection for the amines was about 1 pmol injected for spermine and spermidine and 10 pmol injected for putrescine.

For indole acetic acid (IAA, auxin), aliquots of the supernatants were injected (8 μl) onto a mixed cationic/anionic/reverse phase HPLC column (Imtakt Scherzo SS-C18, 100 × 2 mm, 3 μ particle size and 130 Å pore size) equilibrated in 40% solvent C (water/acetonitrile/formic acid, 97/3/0.1, all by vol) and 60% solvent D (45 mM aqueous ammonium formate/acetonitrile, 65/35, v/v), and eluted (200 μL/min) with an increasing concentration of solvent D (min/%D; 0/60, 5/60, 20/100, 22/60, 30/60). The effluent from the column was directed to the same ESI mass spectrometer as described above, and the time-dependent intensity of the IAA MRM transition was recorded at previously optimized settings [m/z (MH^+^) 176
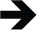
130, fragmentor 45, collision energy 12]. Peak areas for the transition response at the corresponding retention time (10.1 min) were computed as described above. A standard curve was prepared with each experiment from samples containing known concentrations of IAA. Under the prescribed conditions, the limit of detection (LOD) for IAA was about 5 pmol injected, which was about four-fold lower than what could be achieved in the negative ion mode also under previously optimized conditions by monitoring the transition of the (M-H)^−^ ion at m/z 174
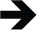
130. Also, the LOD using combined liquid chromatography-MS/MS-MRM (LC/MS/MS-MRM) in either the positive or negative ion mode was significantly lower than what could be achieved by combined gas chromatography/mass spectrometry (GC/MS) in the selected ion-monitoring (SIM) mode (Waters GCT) of the trimethylsilyl derivative.

### Cross-streaking experiments

Fresh samples of each bacterial strain were taken from frozen cultures and grown on either LB minus NaCl, LB, or TY agar for cross streaking (Lertcanawanichakul and Sawangnop, 2008). A single colony from one strain was first streaked vertically down the middle of the plate and 24 h later the second strain was streaked perpendicularly to the first. The order of microbes was changed in each experiment, which was repeated 4 times with 3 or 4 biological replicates. Qualitative data were obtained by photographing the plates daily for 7 days. For the pairs of *S. meliloti* and *B. simplex*, we also performed parallel streaking and overlapping streaking experiments. As before, the order of microbes was changed in each experiment, and the plates were followed for 10 days.

### Plant coinoculation experiments

*Macroptilium atropurpureum* (siratro), *Medicago truncatula* A17, and *Melilotus alba* L. U389 (white sweetclover) seeds were planted in black polyethylene boxes (Really Useful Boxes®) or Magenta® jars (Magenta Corp.). The substrate used for the black boxes was Seramis® (Mars GmbH) and perlite, and for the Magenta jars, a 2:1 mixture of vermiculite and perlite. The substrates were autoclaved and then watered with ¼ strength Hoagland's medium minus nitrogen (Machlis and Torrey, 1956). Prior to planting seeds were scarified for 1 min, soaked in 95% ethanol for 5 min, and then in full-strength commercial bleach for 30–45 min. The conditions for siratro seed sterilization and inoculation with *Bu. tuberum* are detailed in Angus et al. (2013). The imbibed seeds were transferred to the boxes or Magenta jars using sterile tools, and inoculated singly with *Bu. tuberum* (siratro) or *S. meliloti* (*Medicago* and *Melilotus*) and *B. simplex* 30N-5, together and alone. The bacteria were diluted with sterile water to a final OD_600_ nm of 0.1–0.2. The siratro seeds were coinoculated with a 1:1 mixture of *Bu. tuberum* and *B. simplex* 30N-5 whereas the *Melilotus* and *Medicago* seeds were coinoculated with a 1:1 mixture of *S. meliloti* 1021 and *B. simplex* 30N-5. Each Magenta jar or black box was inoculated with 4 mL of the inoculum. Controls were included for all experiments and were used as a phenotypic reference for −N, +N, and no nutrient conditions (water). The control sample size was smaller than the experimental due to space limitation, but the controls consistently gave the same phenotype (see Angus et al., 2013, 2014). The siratro plants were grown in a temperature controlled Conviron growth chamber at 24°C and the *S. meliloti* hosts in a Percival growth cabinet at 21°C. The *Medicago* and *Melilotus* species were harvested 5 weeks after inoculation and the siratro plants 5–6 weeks after inoculation. Each experiment was repeated three times. The plants were photographed, their shoot height and nodule numbers were recorded, and they were then dried (48 h, 65°C) before dry weight measurements were made. Statistical significance of the data was validated using One-way ANOVA with Tukey's post hoc test (**Figure 3A**) and multiple comparison procedure. Jittered boxplot and family-wise error rates (**Figure 3B** and Supplementary Figure 2) were used for assessment (Herberich et al., 2010).

### Genome analysis

#### Selection of strains

**Figure 1:**
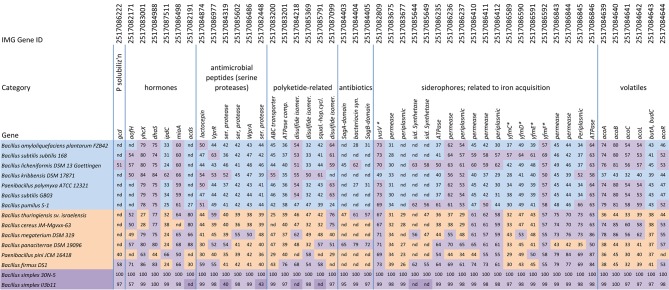
**Homologs of reference set of Plant Growth Promoting (PGP) genes identified in *Bacillus* and *Paenibacillus***. Top row, IMG gene ID number; second row, general categories of PGP genes; third row, PGP genes identified in each *Bacillus* (or *Paenibacillus)* strain (left column) following comparison with the *B. simplex* 30N-5 gene (always 100%) and clustered using the K-means algorithm. The highest sequence identity alignment from blastp searches (% values in cells), sequence identities passing the 50% cutoff (cells highlighted in purple) show 3 clusters: cluster 1 (blue), cluster 2 (orange), and cluster 3 (purple). Genes not detected (nd). ^*^Gene names from *B. megaterium, B. licheniformis*, and *B. amyloliquefaciens* (see text).

Draft and finished genome sequences of several PGPB from the Joint Genome Institute IMG/ER (Markowitz et al., 2012) or from NCBI (http://www.ncbi.nlm.nih.gov) were queried by BLAST (Altschul et al., 1990) using sequences of genes encoding known PGPB traits. Thirteen *bona fide* PGP *Bacillus* and two *Paenibacillus* strains were chosen for comparison against *B. simplex* 30N-5 (Figure 1). The JGI genomes queried included *B. simplex* 30N-5 (permanent draft), *B. simplex* II3b11 (permanent draft), *B. firmus* DS1 (permanent draft), *B. licheniformis* DSM 13^T^/ATCC 14580 (finished), *B. kribbensis* DSM 17871 (permanent draft), *B. megaterium* DSM 319 (finished), *B. amyloliquefaciens* subsp. *plantarum* FZB42^T^ (finished), *B. subtilis* GB03 (permanent draft), *B. subtilis subtilis* 168 (finished), *B. cereus* JM-Mgvxx-63 (permanent draft), *B. thuringiensis* sv. *israelensis* (permanent draft), *Paenibacillus polymyxa* ATCC 12321 (permanent draft), *Paenibacillus pini* JCM16418 (permanent draft), *Pseudomonas fluorescens* strains A506 and CHAO (finished), and *Azospirillum brasilense* FP2 (permanent draft) and *Azospirillum* sp. B510 (permanent draft). *B. simplex* 30N-5 was isolated from the Mildred E. Mathias Botanical Garden at UCLA and strain II3b11 belongs to the Putative Ecotype 9 (Koeppel et al., 2008). It originates from the south facing, hot or “African savannah-like” slope of “Evolution Canyon II” in Nahal Keziv, Israel (Sikorski and Nevo, 2005). In addition, the genome of *B. simplex* strains P558 (Croce et al., 2014) and BA2H3 (Khayi et al., 2015), both from NCBI, were queried when a gene from one or both IMG/ER *B. simplex* strains was missing. Details of the *Bacillus* strains studied for the genomic analysis are found in Table 2.

**Table 2:** **Genomic features of the *B. simplex* 30N-5 genome and comparison with the genomes of other *Bacillus* spp**.

	***B. simplex 30N-5***	***B. simplex II3b11***	***B. thuringiensis sv. israelensis***	***B. pumilus S-1***	***B. kribbensis DSM 17871***	***B. firmus DS1***	***B. amyloliquifaciens pl. FZB42***	***B. licheniformis DSM 13/ATCC 14580***	***B. subtilis GB03***	***B. megaterium DSM 319***
Genome size (bp)	5459036	5582948	5643051	3692073	5054217	4971242	3918589	4222645	3849547	5097447
G+C content (mol%)	40.43	40.27	35.18	41.26	42.93	41.46	46.4	46.19	46.55	38.13
Protein-coding sequences	5288	4841	5349	3786	4914	4922	3693	4196	3705	5100
% of coding region	81.54	70.30	81.27	88.92	81.66	85.02	88.0	88.13	89.61	83.04

#### Homologous gene identification

A curated set of more than 50 PGP genes (47 are displayed in Figure 1), all manually annotated in the *B. simplex* 30N-5 genome, were selected for use as the reference query genes for blastp homolog searches. The genes represented a wide variety of PGP functions. Because several of the genomes investigated have permanent draft status, our analyses of gene homologies occasionally found no matching gene; these “missing” genes are indicated by “n.d.”. We used conservative criteria to compare the protein sequences. The blastp searches were filtered to include alignments with an *e* < 10^−5^, and with a sequence identity of ≥50%, although homologs having smaller percentages and greater *e*-values were present in the other *Bacillus* genomes. *B. simplex* 30N-5 was the reference genome for the comparisons and the value of its gene identities for the comparison was set at 100%. Although both Gram-negative and Gram-positive bacteria were initially screened for PGP traits, the values for the Gram-negative bacteria as well as for the more distantly related Gram-positive species were generally low and hence deleted from the final data set.

We also used BAGEL3 (http://bagel2.molgenrug.nl/) to query the *B. simplex* 30N-5 genome for bacteriocins, and a stand-alone version of ANTIsmash (http://antismash.secondarymetabolites.org/) to search for non-ribosomal peptide synthetases (NRPSs).

#### K-means clustering

The sequence identity matrix (Figure 1) contains 13 *Bacillus* strains and 2 *Paenibacillus* strains (rows) with the highest detected sequence identity for each reference PGP gene displayed in columns. Despite the 50% cutoff sequence identity limit, some sequence identity scores under 50% were also recorded because blastp sequence alignments with low sequence identity may still exhibit homology or contain conserved functional domains. This scheme enabled clustering, using the K-means clustering algorithm, to place *Bacillus* strains into groups that had overall similar profiles of PGP genes either as present or absent. The algorithm was implemented with an objective function to minimize the within-cluster Euclidean distance of the sequence identity vectors (rows) from their assigned clusters.

Our implementation of the K-means algorithm used a two-step iterative algorithm (Lloyd, 1982; Slonim et al., 2013). In the assignment step, the sequence identity vectors (rows) were assigned to the nearest cluster (measured by Euclidean distance between each sequence identity vector and centroid corresponding to each cluster). In the update step, the coordinates of each centroid were updated to the mean of the respectively assigned sequence identity vectors. The maximum number of iterations permitted was 10,000. Initial centroids were randomly assigned to the sequence identity vectors, and 100 random centroid initializations were run. Of the 100 K-means runs, the cluster arrangement minimizing the total Euclidean distance of the sequence identity vectors (rows) from their assigned clusters was retained for visualization (Figure 1).

## Results

### Phylogenies

**Figure 2:**
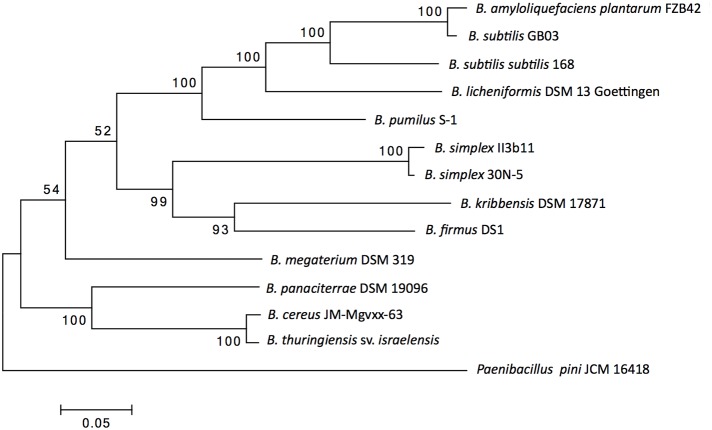
**Phylogenetic tree**. Maximum-likelihood phylogenetic tree based on concatenated gene sequences of five housekeeping genes (*atpD, urvA, rpoB, lepA*, and *recA*). *Paenibacillus pini* JCM 16418 was used as the outgroup. Numbers at branch points indicate bootstrap values (based on 1000 replicates); only those above 50% are indicated. Bar, 0.05 substitutions per nucleotide position.

A concatenated gene Maximum Likelihood phylogeny of the selected strains is shown in Figure 2. The housekeeping genes used for the tree are representative of the differences between the genomes of the species tested, as the most closely related species to *B. simplex* are also those with most similarities found in the PGPB genes studied (see colors in Figure 1). Although the topology within the clade was supported by high bootstrap values, the two subclades in the top part of Figure 2 were supported by a low bootstrap value (52%). One subclade contained *B. amyloliquefaciens* subsp*. plantarum* FZB42, *B. subtilis* GB03, *B. subtilis subtilis* 168, *B. licheniformis* DSM13 Goettingen (ATCC 14580), and *B. pumilus* S-1, whereas the second subclade included the two *B. simplex* strains, and *B. firmus* DS1 and *B. kribbensis* DSM 17871. In this tree, *B. subtilis* GB03 and *B. amyloliquefaciens* subsp. *plantarum* FZB42 clustered together. A not-as-strongly supported branch of the top clade (54% bootstrap support) included *B. megaterium* DSM 319. The clade (bottom part of Figure 2) brought together with strong support, *B. panaciterrae* DSM 19096*, B. cereus* JM-Mgvxx-63, and *B. thuringiensis* sv. *israelensis*. *P. pini* JCM 16418 was the outgroup.

### Pre-coinoculation (cross-streaking) assays

Earlier we reported positive effects on pea growth when *B. simplex* 30N-5 was coinoculated with *R. leguminosarum* bv. *viciae* 128C53 (Schwartz et al., 2013). Before setting up coinoculation experiments with a different set of bacteria, cross-streaking assays were used to detect incompatibility or interference between the nodulating strains, *S. meliloti* 1021 (data not shown) and *Bu. tuberum* STM678 (Supplementary Figure 1A), to be used in the coinoculation study with *B. simplex* 30N-5. No inhibition of growth was found. An additional *Bacillus* strain previously isolated and studied (Schwartz et al., 2013), *B. subtilis* 30VD-1, was also tested in these experiments. *B. subtilis* 30VD-1 inhibited *B. simplex* 30N-5 growth and *vice versa*, suggesting that one or both synthesized bacteriocins or other antimicrobial agents (Supplementary Figure 1A and see later section).

For *S. meliloti*, the results from the initial cross-streak experiments were less clear because although the *S. meliloti* streak was not touching the *B. simplex* one, it was closer to it than the distance observed for the *B. subtilis* and *B. simplex* cross-streaks (Supplementary Figure 1A). When we repeated the experiments by either doing a side-by-side streak or inoculating one strain over the other in a cross pattern, we observed no incompatibility between the two strains (data not shown).

### Coinoculation studies

**Figure 3:**
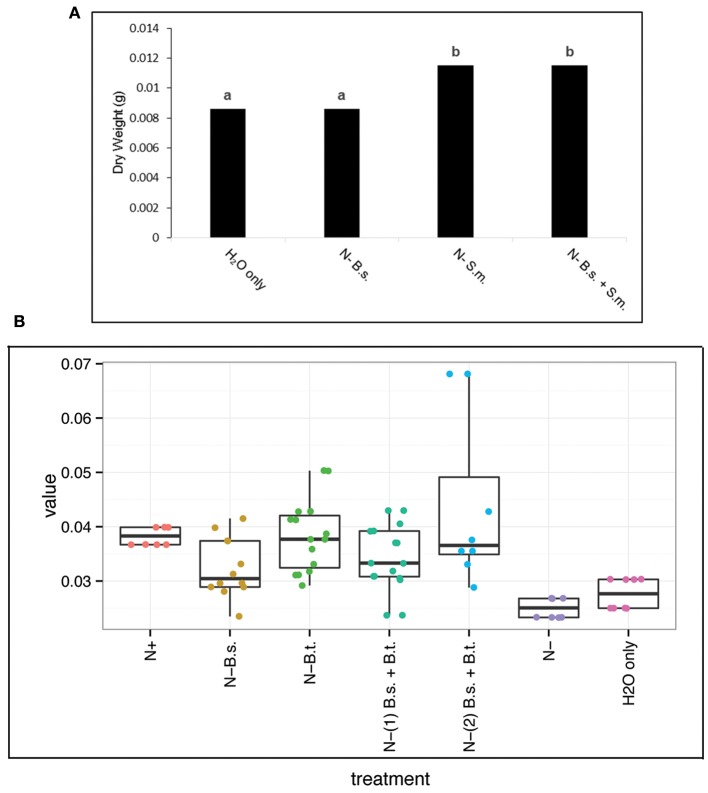
**Biomass measurements of *Melilotus alba* and *Macroptilium atropurpureum* 35 days post inoculation**. **(A)** *Melilotus alba* plants were singly or coinoculated with *Bacilus simplex* (*B.s.*) and *Sinorhizobium meliloti* (*S.m.*); Different letters represent values that differ significantly, *p* < 0.01. **(B)** Jittered boxplot. *Macroptilium atropurpureum* plants were singly or coinoculated with *Bacillus simplex* (*B.s.*) and *Burkholderia tuberum* (*B.t*). The first coinoculation (1) introduced both bacteria species at the same time, whereas the second (2) was inoculated with *B.s.* first followed by *B.t.* inoculation 5 days later. Harvesting was performed as described in Methods. Boxes indicate minimum, maximum, 1st and 3rd quartiles and the median value.

Because *B. simplex* 30N-5 demonstrated a positive effect on both plant growth and rhizobial nodulation on pea (Schwartz et al., 2013), we tested whether or not this was a general phenomenon by coinoculating *B. simplex* 30N-5 and *S. meliloti* Rm1021 onto roots of *M. truncatula* and *M. alba*. In contrast to our previous results with pea, *M. alba* exhibited no significant growth enhancement when inoculated with *B. simplex* alone over the uninoculated control (Figure 3A). Although shoot height and nodule number were measured for all the conditions examined, no statistical significance was observed when the experimental treatments were compared with their respected controls (data not shown). Moreover, when single inoculations with *S. meliloti* and coinoculations with both strains were compared, the treatments (measured as dry weight increase) did not differ from each other although both were statistically different from the uninoculated and *B. simplex* alone-inoculated plants (Figure 3A). *M. truncatula* exhibited a similar response (data not shown). Overall, we found that dry weight increases were a more reliable measurement of plant biomass accumulation than any other parameters (see next section).

Siratro plants were coinoculated with *B. simplex* and *Bu. tuberum*; the latter nodulates siratro effectively (Angus et al., 2013). In contrast to the *S. meliloti* host plants, simultaneous coinoculation with *B. simplex* and *Bu. tuberum*, or coinoculation with *B. simplex* first and then *Bu. tuberum* 5 days later resulted in significant changes over the controls and were comparable to or better than the +N control. The siratro plants inoculated with *B. simplex* alone also exhibited an increase in dry weight over the −N control and were comparable to the +N control (Figure 3B, Supplementary Figure 2).

### Nutrient acquisition

Although *B. simplex* was isolated on a solidified N-free medium, it is not a diazotroph because it lacks *nifH*, a structural gene essential for nitrogenase function (Schwartz et al., 2013). In an N-free liquid medium, *B*. *simplex* 30N-5 ceased growing unless the medium was supplemented with 1-aminocyclopropane-1-carboxylate (ACC), which is broken down into 2-oxobutanoate and ammonia; the latter sustained bacterial growth for a short time. This finding suggested that *B. simplex* had *acdS* activity (see later section).

#### Phosphate solubilization

*B. simplex* 30N-5 effectively solubilized mineralized phosphate as measured by activity on PVK plates (Schwartz et al., 2013). Although we detected a gene encoding a soluble quinoprotein glucose/sorbosone dehydrogenase, which is important for gluconic acid production (de Werra et al., 2009) (Figure 1, column 1), no additional genes involved in the breakdown of either inorganic or organic phosphates were found. However, the putative *B. simplex* (*gcd*) gene was not overly similar to the comparable genes in either *B. licheniformis* (51%) or *B. firmus* (58%) (Figure 1). We were unable to detect an equivalent gene in the other PGPB strains. Species of *B. amyloliquefaciens* (Kim et al., 1998), *B. licheniformis* (Tye et al., 2002), and *B. subtilis* (Kerovuo et al., 1998) have been reported to produce phytase (myo-inositol-hexaphosphate 3-phosphohydrolase), which degrades organic phosphates. A phytase gene was not detected in the *B. simplex* strains.

#### Siderophores

Siderophores secreted by bacteria also support the development and growth of plants by helping them sequester iron from the environment. Previously, we showed that *B. simplex* 30N-5 exhibited a positive reaction in a CAS assay, which detects siderophore activity (Schwartz et al., 2013). We identified several siderophore operons in *B. simplex* 30N-5 (Figure 1). The iron-dicitrate transporter genes were similar to *yfmCDEF* of *B. megaterium* and several other bacilli, whereas the iron-compound transport system had genes conserved with *yfiZ* and *yfiY* of *B. licheniformis* as well as with *B. amyloliquefaciens* genes.

#### Production of volatiles

Many PGPB emit volatiles that positively enhance growth, e.g., *B. amyloliquefaciens* subsp. *plantarum* FZB42 and *B. subtilis* GB03. The latter was reported to acidify the rhizosphere of Arabidopsis in response to volatiles (Zhang et al., 2007), which may help in phosphate solubilization. The most commonly studied volatiles are acetoin (Xiao and Xu, 2007), and 2,3-butanediol, which is known to be involved in Arabidopsis defense induction (Ryu et al., 2003). The Voges-Proskauer test was used to demonstrate acetoin synthesis as well as the potential for production of 2,3-butanediol (Xiao and Xu, 2007) by means of a colorimetric reaction. We used this test on a number of *Bacillus* strains known to produce volatiles and included *B. simplex* 30N-5 along with two additional strains of *B. simplex* (Kaplan et al., 2013) for the analysis. *Escherichia coli* was the negative control. Although the known PGPB strains tested positive for acetoin, including *P. polymyxa* NRRL B-4317, the three *B. simplex* strains were negative even after a long incubation period (Table 3).

**Table 3:** **Voges-Proskauer test results for selected strains**.

**Strain**	**30′**	**1 h**
*E. coli* HB101^*^	−	−
*B. amyloliquefaciens plantarum* FZB42	+	+
*B. cereus* 26a1	−	−
*B. licheniformis* DSM 13/ATCC 14580	+	+
*B. simplex* 237	−	−
*B. simplex* 11	−	−
*B. simplex* 30N-5	−	−
*B. subtilis* GB03	+	+
*B. subtilis* 60b4^**^	+	+
*P. polymyxa* NRRL B-4317	+	+

* Negative control

** Positive control.

Measurements were taken 30 min and 1 h after the addition of the colorimetric reagents.

Genes from *B. amyloliquefaciens* subsp. *plantarum* FZB42 were used to search for sequences in the *B. simplex* genome that could encode proteins for acetoin synthesis. We detected five genes, several *aco* genes, as well as one encoding *alsS*, which is important for acetolactate synthesis (Xiao and Xu, 2007). However, no gene for *alsD*, which encodes an alpha-acetolactate decarboxylase, was detected although it was present in the reference PGPB genomes. Also, a *butA*/*budC* gene was detected in the *aco* operon and showed greater than 95% identity to the other *B. simplex* strain (II3b11) (Figure 1). The *butA/budC* gene encodes meso-butanediol dehydrogenase.

### Root colonization and growth promotion factors

#### Motility

**Figure 4:**
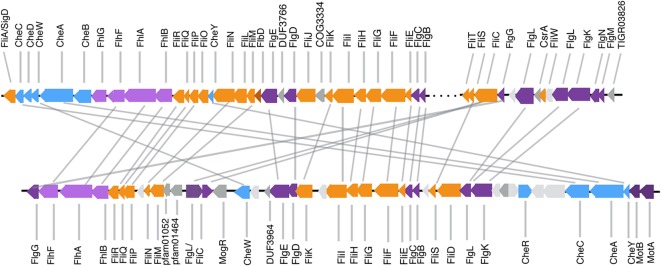
**Comparison of flagellar open reading frame (ORF) clusters**. The top cluster is from *B. simplex* 30N-5, which consists of two unlinked regions. The bottom cluster is from *B. cereus* JM-Mgvxx-63 where most of the genes related to *B. simplex* are within a single region on the chromosome.

In response to root exudates, many bacteria migrate toward root surfaces and colonize roots. In *B. amyloliquefaciens* subsp. *plantarum* FZB42 and other *Bacillus* species, a number of genes are expressed (Chen et al., 2007), including flagellar genes (De Weger et al., 1987; Croes et al., 1993). In the *B. simplex* genome, flagellar and chemotaxis genes are located in two apparently unlinked areas (Figure 4). A similar arrangement exists for other PGPB *Bacillus* strains, such as *B. firmus* DS1, *B. kribbensis* DSM 17871, *B. megaterium* DSM 319 (Supplementary Figure 2), and the two *Paenibacillus* species included in our analysis (data not shown). However, a major difference in arrangement was observed in *B. cereus*, which contains strains that can be either beneficial or pathogenic (Bottone, 2010). For example, five chemotaxis-associated genes (*cheABCD* and *W*) within the flagellar gene operon are conserved among *B. simplex* and related PGP strains (Figure 4, Supplementary Figure 3). However, the chemotaxis genes, *cheB* and *cheD*, were not detected within the flagellar gene region of *B. cereus* JM-Mgvxx-63, which differs in organization (Figure 4). In addition, the flagellar genes are not highly related. For example, the *flhF* genes of *B. simplex* 30N-5 and *B. cereus* JM-Mgvxx-63 are 36% identical, but only 24% DNA identity is observed when the two *fliS* genes were compared.

Many PGPB, e.g., alpha-rhizobia (Amaya-Gómez et al., 2015) and *Bacillus* spp. (Dietel et al., 2013), swarm as well as swim prior to colonization. *B. simplex* 30N-5 cells swarm on 0.8% agar suggesting that genes for this behavior are present and expressed. The *swrC* gene shares 100% identity among the *B. simplex* strains. Although originally annotated as a cation/multidrug efflux pump, it is orthologous and 61 and 57% identical to genes annotated as *swrC* in *B. thuringiensis* and *B. amyloliquefaciens*, respectively.

### Plant growth-promoting traits: hormones

One of the most prominent features of PGPB is their ability to produce compounds that directly influence plant growth, e.g., the phytohormones. PGPB also synthesize gene products that affect plant growth in a more indirect way.

#### Auxin

Many bacteria are known to synthesize auxin (involved in lateral root proliferation), and at least five tryptophan-dependent or tryptophan-independent biosynthetic pathways are employed by bacteria to synthesize auxin (Patten and Glick, 1996; Spaepen and Vanderleyden, 2011). Because we obtained positive results with the Salkowski test following the addition of tryptophan (Schwartz et al., 2013), we hypothesized that the genes for auxin synthesis might be present in the genome of this species.

One of the most studied of the auxin biosynthetic pathways includes the genes encoding IAA monooxygenase (*iaaM*) and indole acetamide hydrolyase (*iaaH*), found in the gall-forming *Agrobacterium tumefaciens* and *Pseudomonas savastonoi*, but these genes were not detected in the *B. simplex* 30N-5 genome. However, putative *ipdC* genes were identified in the *B. simplex* strains with greater than 97% DNA identity among them, following a query with an indole pyruvate carboxylase gene from *Enterobacter cloacae* (Koga, 1995). Although < 50% DNA identity to *ipdC* from several other PGPB was observed (Figure 1), *ipdC* genes in *B. kribbensis* and *P. pini* were 62 and 66% identical in DNA sequence, respectively, and orthologous to the *B. simplex* gene. A gene encoding a putative indole-3-acetaldehyde dehydrogenase, which is involved in tryptophan-dependent IAA synthesis and is the last step in the pathway, was also detected in *B. simplex* 30N-5. The gene is ca. 75% identical to *dhaS* of several bacilli, and has >84% identity to genes in *B. firmus* DS1 and *B. kribbensis* DSM 17,871.

Other auxin-related genes were also found in *B. simplex* 30N-5. A gene orthologous to *aofH*, which codes for an indole-3-acetic oxidase [suggesting that tryptamine, rather than tryptophan, is converted to indole-3-acetaldehyde (IAAld)], was uncovered using AZL.b03560 from *Azospirillum* sp. B510 (Wisniewski-Dyé et al., 2011) to query the *B. simplex* genome. Although the different *B. simplex* strains have almost identical *aofH* gene sequences, this gene is not well conserved with genes from other bacilli with the exception of *B. firmus* DS1 (71% identity) (Figure 1). Lastly, a *B. simplex* gene with 79% DNA identity and orthologous to a predicted nitrilase, the *yhcX* gene in *B. amyloliquefaciens*, was detected in the *B. simplex* 30N-5 genome. A similar gene identity was observed for many of the other PGP bacilli (Figure 1).

The lack of a complete pathway and the low identity for *ipdC* made us question whether IAA is actually synthesized by *B. simplex.* To address this question, we performed a chemical analysis. No signals for IAA were found in the LC/MS/MS-MRM assay for this compound. With a limit of detection of about 5 pmol injected, it is concluded the concentration of this hormone, if present, is less than 174 pmol/10^9^ cells.

#### Phenylacetic acid

In *Azospirillum brasilense*, IpdC is also involved in the production of phenylacetic acid (PAA), which has weak auxin activity and is also antimicrobial against both bacteria and fungi (Somers et al., 2005). As in *Azospirillum*, the *B. simplex* genome has the *paa* operon (data not shown), which is important for the degradation of PAA.

#### Cytokinin

Many PGP bacilli have been reported to produce cytokinins, but few cytokinin biosynthetic genes have been detected (see Vacheron et al., 2013). Querying various *Bacillus* genomes with *tzs* (trans-zeatin synthase) from *A. tumefaciens*, where it is required for tumor formation, yielded no hits. On the other hand, *miaA*, which encodes tRNA dimethylallyltransferase that removes a zeatin precursor from tRNA, is common among the PGP bacilli, including *B. simplex*.

#### Polyamines

Many bacteria produce polyamines such as spermine, spermidine, and putrescine, which in *B. subtilis* OKB105 have PGP properties (Xie et al., 2014). A number of genes involved in polyamine synthesis (Sekowska et al., 1998) were detected in the *B. simplex* 30N-5 genome including *speA*, which results in agmatine synthesis; *speB*, putrescine synthesis; and *speE* and *speD*, which encode the stages for spermidine synthesis. Also, *metK*, responsible for the conversion of methionine to S-adenosyl-methionine, was detected (data not shown). Many of these were ≤ 80% identical to genes in the PGP bacilli (*speD*), whereas others, e.g., *speE*, are less similar ( < 50%) albeit orthologous. Genes for various binding proteins, permeases, and transporters for polyamines are also present in the genome of *B. simplex* 30N-5.

**Figure 5:**
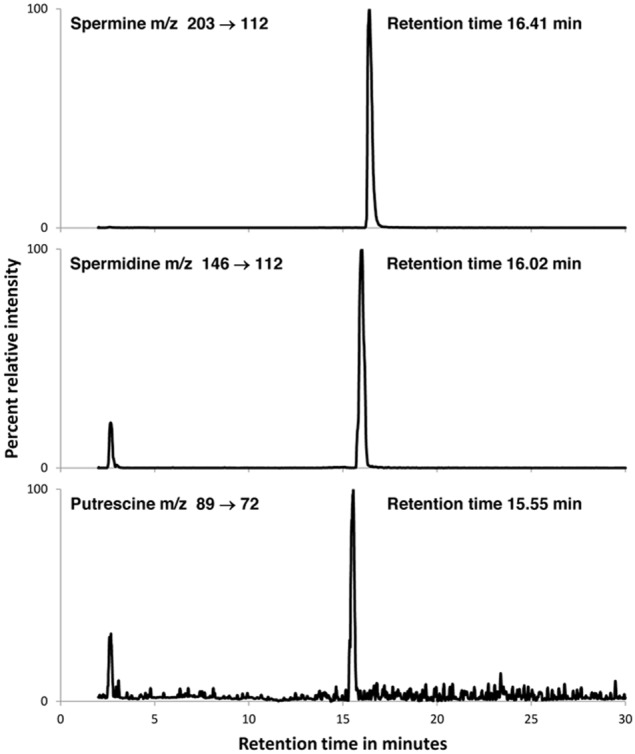
**LC/MS/MS-MRM traces for the TCA extract of *B. simplex* 30N-5**. Peaks for spermine (top), spermidine (middle) and putrescine (bottom) are shown. Samples were prepared and analyzed as described in Methods. Co-chromatography experiments in which the authentic compounds were added to the bacterial extract showed single peaks for each trace with appropriate augmentation of the peak areas. A quantitative summary of the results is presented in Table 4.

Clear strong signals were obtained for cell lysates prepared in either TCA or TFA showing the presence of significant quantities of spermine, spermidine, and putrescine in all samples examined (Figure 5). Verification of the assignments was made with co-chromatography experiments in which known amounts of authentic standards were added to cell lysate samples prior to LC/MS/MS-MRM. In these experiments, single peaks for each amine were obtained for the spiked samples, with appropriate area and intensity enhancement of the signals. The signals were slightly more intense for spermine and spermidine when TFA was used compared to TCA during cell homogenization (1.6- and 1.2-fold, respectively), and slightly more intense for putrescine when TCA compared to TFA was used (2.8-fold) (Table 4). The MRM chromatograms from methanol extracts were less clear with peaks at other retention times and significantly less intense peaks for the polyamines. Quantitation based on external standards shows spermine, spermidine and putrescine concentrations in the range of 333, 222, and 2.2 nmol/10^9^ cells, respectively, although for more precise measurements, the work requires repeating using an internal standard to correct for losses during extraction. To this end, it was noted that the cell extracts did not contain any detectable amount of hexamethylediamine (MRM transition (MH^+^) m/z 117
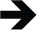
100) that could be used for this purpose.

**Table 4:** **The concentrations of spermine, spermidine, and putrescine in methanol, TFA, and TCA extracts of *B. simplex* 30N-5 measured by LC/MS/MS-MRM using external standards**.

**Sample**	**nmol/sample**	**nmol/sample**	**nmol/sample**
30N-5 methanol extract 1	0.92	1.46	1.09
30N-5 methanol extract 2	1.03	5.38	1.18
30N-5 TFA extract 1	613.75	355.15	2.02
30N-5 TFA extract 2	622.45	462.71	1.18
30N-5 TCA extract 1	400.68	388.64	5.28
30N-5 TCA extract 2	380.60	312.82	3.53

#### AcdS

Ethylene is inhibitory to root development and also induces plant defense response pathways. We detected a sequence in *B. simplex* 30N-5 that is similar to genes annotated as *acdS* in *B. thuringiensis* and in *B. cereus* JM-Mgvxx-63 (80%) and as D-cysteine desulfhydrase in *B. panaciterrae* DSM 19096 (88%) (Figure 1). The comparable genes for the two additional *B. simplex* strains available at NCBI were annotated as a cytochrome C biogenesis protein/D-cysteine desulfhydrase. These proteins are part of the PLP-dependent ACC family. However, genes homologous to this sequence were not detected in the typical PGPB group (blue group; Figure 1).

### Antibiotics and related compounds

Bacteria synthesize a number of compounds that contribute to their survival in the rhizosphere. *B. subtilis* has been a paradigm for studying these antimicrobial compounds, which fall into two major classes: those synthesized on ribosomes (e.g., bacteriocins and lantibiotics) and post-translationally modified and those produced on large multienzymes known as Nonribosomal Peptide Synthetases (NRPSs), e.g., iturin and fengycin (Stein, 2005).

#### Bacteriocins

Many nonpathogenic bacteria produce bacteriocins, molecules used to compete with closely related bacteria, and many classes of these antimicrobial peptides are known. We scanned the *B. simplex* genome for genes potentially encoding bacteriocins and found three candidates, but genes comparable to those in *B. simplex* were not detected (n.d.) in any of the other PGP bacilli (Figure 1). The highest DNA sequence identity of genes encoding proteins for bacteriocin synthesis (Figure 1) was to the sequences found in *B. panaciterrae*. The highest DNA sequence identity of the bacteriocin biosynthesis gene, based on amino acid sequence, was 79% (Figure 1). These same gene sequences were picked up using the BAGEL3 website and a gene map is depicted in Supplementary Figure 1B.

Another protein with 99 and 100% identity to the two *B. simplex* strains in NCBI (*B. simplex* P558, CEG34010.l; *B. simplex* BA243, WP 034090.1, respectively) was also found. It matched to a protein described as a colicin V production protein. Although the gene neighborhoods were well conserved among the *Bacillus* species in Figure 2, the percentage DNA identity was 70% or lower (data not shown).

#### Additional secondary metabolites

Many PGPB synthesize diverse secondary metabolites, which have antibiotic activity, including lantibiotics, nonribosomally synthesized peptides, and polyketides. For example, subtilin is a 32-amino acid pentacyclic lantibiotic produced by *B. subtilis* (Stein, 2005). Although several subtilisin-like serine protease (AprE-like) genes are present in the *B. simplex* genome as well as proteins involved in subtilin processing (WprA and Vpr-like), no evidence was found in the *B. simplex* genome for the presence of genes similar to *spaS* and *spaBTC*, the subtilin structural genes and the genes promoting subtilin expression, respectively, nor to the genes *spaIFEG*, which confer immunity. Similarly, we saw no matches to genes encoding lantibiotic-like peptides such as sublanchin or subtilisin A produced by *B. subtilis*.

We looked for, but did not find, genes for the synthesis of nonribosomally synthesized peptide antibiotics in *B. simplex*, such as surfactin (see next section), iturin, or bacillomycin or for antimicrobial polyketides such as macrolactin, bacillaene, or difficidin, which are found in many PGPB *Bacillus* strains.

#### Other nonribosomal peptide synthetase (NRPS) products

Genes were found for the synthesis of koranimine, a cyclic imine. The genes involved are: *korA, korB, korC, korD*, as well as genes encoding a phosphopantetheinyl transferase (*kfp*) and a type II thioesterase (*korTE*) (Supplementary Figure 4). These genes had been detected earlier in an environmental *Bacillus* strain (NK2003) using a proteomics-based approach (Evans et al., 2011). We found them using a *B. amyloliquefaciens* subsp*. plantarum* FZB42 gene sequence (*srfAA*) in a blastp search, and although only 34% sequence identity was found between *korA* and *srfAA*, their amino acid adenylation domains were highly conserved (89.7%). Genome analysis led to the discovery of an orthologous gene in *Bacillus* sp. NK2003, which was annotated as a nonribosomal peptide synthetase. Koranimine synthetic genes were also found in the *B. simplex* II3b11 genome, each gene having greater than 95% DNA sequence identity to the *kor* genes of *B. simplex* 30N-5. An amino acid adenylation domain sequence that lines up with the middle part of *korC* was found in another part of the *B. simplex* genome (data not shown).

Although we utilized a gene encoding a surfactin to uncover the *kor* genes, we could not find any genes for surfactin production itself or any other NRPS-produced metabolites in *B. simplex*. Moreover, using a modification of a published procedure of the drop-collapsing assay (Kuiper et al., 2004) to determine surfactant activity, we found none of the three *B. simplex* strains tested exhibited a change in the diameter of the drops due to decrease in surface tension of the droplet (data not shown), suggesting that *B. simplex* 30N-5 lacks surfactant activity.

We also detected the polyketides described above using the ANTIsmash webserver. In addition, evidence for a gene encoding squalene synthetase and highly conserved with the genes of other *B. simplex* strains, but not found in the PGPB bacilli. An orthologous gene with 60 and 57.5% DNA identity was detected in *B. panaciterrae* and *B. megaterium*, respectively (data not shown). Similarly, a gene encoding chalcone synthase that is orthologous and 63–61% identical to genes in *B. kribbensis* and *B. firmus*, respectively, was found using ANTIsmash. The genes of the PGPB bacilli are orthologous and 51% identical to the *B. simplex* gene (data not shown).

### Other pathways

#### Vitamins

More than one-third of the bacteria that have been sequenced possess genes for cobalamin (vitamin B12) synthesis (Raux et al., 2000), including *B. simplex* 30N-5. The *B. simplex* 30N-5 and the other *B. simplex* genomes also contain genes for riboflavin (vitamin B2) synthesis as previously described for *B. subtilis* (Stahmann et al., 2000). Riboflavin subunit alpha (*ribF*) was also found in all genomes. Similarly, the menaquinone (vitamin K2) pathway genes found in *B. subtilis* (Sato et al., 2001) were detected in the genomes of the *B. simplex* strains (chorismate synthase, isochorismate synthase, demethylmenaquinone methyltransferase, and 2-heptaprenyl-1,4-naphthoquinone methyltransferase).

#### Protein secretion systems

Gram-positive bacteria secrete proteins usually by translocation across the single membrane by the Sec pathway or via the two-arginine (Tat) pathway. *B. simplex* also possesses the genes, with a 99% identity, *tatA, tatC* and a third gene from the same family. In addition, a specialized secretion system, which is responsible for protein translocation across both the membrane and the cell wall, called a type VII secretion system (Tseng et al., 2009), was detected in the *B. simplex* 30N-5 genome.

## Discussion

*B. simplex* has been shown in a number of reports to be an effective PGPB (Ertruk et al., 2010; Hassen and Labuschagne, 2010; Schwartz et al., 2013). In this report, we show that *B. simplex* strains 30N-5 and II3b11 are phylogenetically and genetically different from the known PGPB bacilli. Based on an analysis of 5 different housekeeping genes, they cluster in a separate subclade from most PGPB bacilli and their PGP-related genes. We thus placed them into a group separate from other PGPB (purple, Figure 1). Because interest in this species as a PGPB species and producer of novel enzymes has been increasing (Velivelli et al., 2015; Venkatachalam et al., 2015), we undertook a detailed investigation of the potential of *B. simplex* 30N-5 to act as a PGPB.

A survey of the three legume hosts used in the coinoculation studies suggests that *B. simplex* may behave differently among plant species. In our study on pea, we observed that simultaneous inoculation of *B. simplex* and *R. leguminosarum* bv. *viciae* resulted in an enhancement of nodulation and plant dry weights (Schwartz et al., 2013). In contrast, coinoculation of *S. meliloti* Rm1021 and *B. simplex* did not produce a significantly different dry weight measurement for either *Melilotus alba* or *Medicago truncatula* compared to the single inoculation. Moreover, *B. simplex* alone did not enhance the growth of the *S. meliloti* hosts compared to pea (Figure 3A).

In contrast, siratro responded positively to single inoculation with *B. simplex* and showed an increase in dry weight, as we had observed for pea (Schwartz et al., 2013). Whether or not this difference between the *S. meliloti* hosts and siratro and pea in response is a consequence of having smaller seeds vs. larger seeds is not known. The larger-seeded legumes contain more stored N, which results in a protracted growth response under N-deficient conditions. In addition, some PGPB strains appear to exhibit host specificity toward different plants (Kloepper, 1996). We are investigating these possibilities further.

Flagella are important for root colonization via cell motility, swarming, and biofilm formation. We found that *B. simplex* and the PGPB bacilli of the blue group (Figure 1) have an identical flagellar gene arrangement (Figure 4, Supplementary Figure 3). In contrast, members of the orange group (Figure 1), *B. thuringiensis* and *B. cereus*, have a different flagellar arrangement (Figure 4). It is not known if this difference is significant. In our previous studies of plant-associated *Burkholderia* species, the arrangement of the flagella genes between the plant-associated *Burkholderia* species and the mammalian and opportunistic pathogens was also dissimilar (Angus et al., 2014). In *Burkholderia*, the flagellar genes were linked together on a chromosome in the plant-associated species whereas they were located in different parts of the genome in the pathogen-clade. Flagella from pathogenic species are well known for triggering induced systemic resistance (ISR) in numerous plant species, but information about whether flagella from beneficial bacteria, especially from PGPB *Bacillus* spp., affect host responses is not available.

Volatiles are also important for inducing a systemic response. Although several genes associated with the acetoin pathway are present in *B. simplex* 30N-5, this strain did not produce detectable quantities of acetoin based on the Voges-Proskauer test. We observed that none of the *B. simplex* genomes that have been sequenced contain the *alsD* gene, whereas the genomes of the typical PGPB bacilli do. Because AlsD is missing, alpha-acetolactate, which is unstable following synthesis by AlsS via the condensation of two pyruvate molecules, cannot be converted to 3-hydroxy-2-butanone (acetoin), the precursor of 2,3-butanediol (Xiao and Xu, 2007). *B. simplex* M3-4, which has a positive effect on potato tuber yields, was also found to be negative for the Voges-Proskauer test (Velivelli et al., 2015). Nonetheless, this strain produces volatiles, such as 2-hexen-1-ol, 2,5-dimethylpyrazine, and several others, which inhibit *Rhizoctonia solani* growth. Thus, *B. simplex* strains are effective PGPB even though they do not emit 2,3-butanediol (this work; Velivelli et al., 2015). Future studies will investigate whether *B. simplex* 30N-5 produces similar volatiles.

Plant growth promotion frequently results from the action of hormones, and auxin synthesis is a common trait that is associated with PGPB bacilli. In it, tryptophan is converted to IPA by L-tryptophan aminotransferase (Patten and Glick, 1996). Although several aminotransferases were detected in the *B. simplex* genome, none could be specifically designated as this enzyme or as tryptophan transferase. In addition, even though genes were found for *ipdC* (indole pyruvic acid carboxylase), *dhaS* (indole-3-acetaldehyde dehydrogenase), *aofH* (indole-3-acetaldehyde oxidase), and *yhcX* (a nitrilase) based on similarities to other *Bacillus* spp., the gene identities were low except for *dhaS* and *yhx* (Figure 1). Using a sensitive and specific LC/MS/MS-MRM assay, we could not detect IAA in cell culture homogenates, further supporting the absence of an active biosynthetic pathway for this hormone in *B. simplex* 30N-5. These results lead us to propose that the commonly used Salkowski test is not definitive for the synthesis of auxin by bacteria.

Polyamines are also PGP compounds. Studies by Xie et al. (2014) showed that mutations in *yecA* (a permease) and *speB* (encoding one of the first steps in the conversion of agmatine to putrescine and then spermidine) eliminated the PGP activities of *B. subtilis* OKB105, e.g., root elongation. Reverse phase HPLC with UV detection of chemically-derivatized samples detected spermidine in the OKB105 culture filtrate (Xie et al., 2014). Our data using a more specific and sensitive assay show that all three polyamines are present in the culture medium of *B. simplex* 30N-5, and indeed based on the genome information, the entire pathway for polyamine production is present.

The enzyme *acdS* is thought to improve plant growth by interfering with ethylene formation. It does this by deaminating 1-aminocyclopropane-1-carboxylic acid (ACC), a direct precursor to ethylene production. Previously, we cloned a potential ACC deaminase gene from *B. simplex* 30N-5 by using *acdS* primers and found a sequence that was closely related to a gene encoding a pyridoxal phosphate-dependent enzyme. Experimental evidence will be needed to determine if this gene product has AcdS activity, but based on the fact that ACC deaminase is a member of the above protein family, it might be *acdS*. Nevertheless, because this gene in the *B. simplex* genomes (P558 and BA2H3) available at NCBI was annotated as a cytochrome C biogenesis protein/cysteine desulfhydrase, we cannot be completely certain that it encodes AcdS. However, ethylene synthesis is inhibited in plant root cells in response to *B. subtilis* OKB105 in response to polyamine synthesis (Xie et al., 2014), suggesting that polyamines may be an additional or alternative mechanism used by certain bacilli for reducing ethylene content in plants.

Genes encoding surfactin or related polyketides were not detected in *B. simplex* 30N-5, but the entire pathway for the synthesis of koranimine, a newly identified NRPS-synthesized peptide (Evans et al., 2011) was found. Although the function of this compound is unknown, cyclic imines are well known marine-based bacterial compounds that accumulate in crustaceans and fish possibly for defense purposes because of their toxicity to predators when ingested (Otero et al., 2011). Hence, the possibility exists that koranimine may play an antibiotic role in its interactions with other microbes and in protecting the plant. Again, additional studies are needed.

In summary, *B. simplex* 30N-5 exhibits potentially novel PGPB properties that are shared, but also dissimilar from some of the more commonly studied PGP bacilli. To be certain that this microbe has no deleterious effects on plants, we are testing it and related strains on both legumes and nonlegumes as well as on *Caenorhabditis elegans* to determine its lack of virulence. Published data showed that *B. simplex* 237 did not have a detrimental effect on *C. elegans* (Angus et al., 2014), and our preliminary results with *B. simplex* 30N-5 demonstrate no toxic effects on nematodes or onions (M. Arrabit and A.M. Hirsch, unpubl.). Also, based on our investigations of the genome, no obvious virulence genes are observed. Thus, *B. simplex* 30N-5 may be an excellent candidate to be added to the group of beneficial bacilli that help plants grow and survive under sustainable agriculture conditions.

## Author contributions

MM, PM, KF, and AH designed and conducted experiments, and wrote the manuscript. ES and AH conceived the work, and ES made critical revisions to the manuscript. ES, WV, PM, KC, TA, JS, and AH acquired the genomics data, and ST and AH interpreted it. MM, PM, KF, TI, LH, MC, TS, NF, and JV acquired experimental data. MM and JS did the statistical analyses. All authors read and approved the final manuscript.

### Conflict of interest statement

The authors declare that the research was conducted in the absence of any commercial or financial relationships that could be construed as a potential conflict of interest.
