# Supplementary material for: Mining the phytomicrobiome to understand how bacterial coinoculations enhance plant growth
Source: Front Plant Sci. 2015 Sep 24;6:784. doi: 10.3389/fpls.2015.00784 (PMC4585168; doi:10.3389/fpls.2015.00784)
Supplement: Supplementary file 1 [file Table1.DOC]

Table S1. IMG ID numbers for the genes used in the concatenated phylogenetic tree.

|  |  |  |  |  |  |
| --- | --- | --- | --- | --- | --- |
| Gene name | *atpD* | *uvrA* | *rpoB* | *lepA* | *recA* |
| *B. simplex* 30N-5 | 2517086969 | 2517086404 | 2517085912 | 2517085308 | 2517082523 |
| *B. simplex* II3b11 | 2503914721 | 2503914613 | 2503916574 | 2503913091 | 2503915812 |
| *B. thuringiensis sv. israelensis* | 2579623636 | 2579624149 | 2579623725 | 2579622113 | 2579620915 |
| *B. megaterium* DSM 319 | 646761375 | 646761309 | 646756391 | 646760796 | 646760348 |
| *B. subtilis* GB03 | 2570683326 | 2570683848 | 2570683126 | 2570685657 | 2570684645 |
| *B. kribbensis* DSM 17871 | 2524546190 | 2524543830 | 2524547502 | 2524546939 | 2524546297 |
| *B. cereus* JM-Mgvxx-63 | 2576354772 | 2576354621 | 2576349883 | 2576353901 | 2576353253 |
| *B. pumilus* S-1 | 2549737025 | 2549737567 | 2549738270 | 2549736883 | 2549740009 |
| *B. licheniformis* DSM 13 Goettingen | 639313039 | 639312874 | 639309325 | 639311887 | 639311083 |
| *B. panaciterrae* DSM 19096 | 2525229252 | 2525229338 | 2525229524 | 2525226918 | 2525226678 |
| *B. amyloliquefaciens plantarum* FZB42 | 640887442 | 640887276 | 640884182 | 640886427 | 640885725 |
| *B. firmus* DS1 | 2569329898 | 2569330571 | 2569332964 | 2569332248 | 2569331893 |
| *B. subtilis subtilis* 168 | 646320306 | 646320141 | 646316534 | 646319124 | 646318232 |
| *P. pini* JCM 16418 | 2563930147 | 2563930263 | 2563930179 | 2563926895 | 2563929705 |
