# Supplementary material for: Mining the phytomicrobiome to understand how bacterial coinoculations enhance plant growth
Source: Front Plant Sci. 2015 Sep 24;6:784. doi: 10.3389/fpls.2015.00784 (PMC4585168; doi:10.3389/fpls.2015.00784)
Supplement: Supplementary file 2 [file DataSheet1.DOCX]

Supplementary Figure 1. (A) Cross-streaking experiments. The x-axis corresponds to the vertical streak and the y-axis corresponds to the horizontal streaks. The vertical streak was streaked first followed by the horizontal streak after 24 h. Left side: *B. simplex* 30N vs. *B. subtilis* 30VD-1. Top, *B. simplex* first; bottom, *B. subtilis* first. Growth inhibition in observed in either orientation. Right side: *B. simplex* 30N vs. *Bu. tuberum* STM678. Top, *B. simplex* first; bottom, *Bu. tuberum* first. The plates were monitored for 7 days. (B) Gene map derived from BAGEL3 showing the three genes involved in the production of bacteriocin in *B. simplex* 30N-5.

Supplementary Figure 2. Graphical visualization of the multiple comparison of treatments using biomass measurements for siratro. The filled black circles indicate the point estimator of the difference between the mean of the groups. 95% confidence intervals are indicated by horizontal bars and parentheses.

Supplementary Figure 3. Alignment of the flagellar region of the *B. simplex* strains with the comparable open reading frames in other PGPB bacilli using IMG/ER (Markowitz *et al.* 2012). *Bacillus* sp. FJAT-14578 was isolated from a soil in China and recently identified as *Bacillus fengquiensis* FJAT-14578 (Zhao *et al*. 2014). The ORF indicated in red is *fliA*/*sigD*.

Markowitz, V.M., Chen, I.M., Palaniappan, K., Chu, K., Szeto, E., Grechkin, Y., Ratner, A., Jacob, B., Huang, J., Williams, P., Huntemann, M., Anderson, I., Mavromatis, K., Ivanova, N.N., Kyrpides, N.C. (2012). IMG: the integrated microbial genomes database and comparative analysis system. *Nucl. Acids Res.* 40, D115–D122. doi:10.1093/nar/gkr1044.

Zhao, F., Feng, Y.Z., Chen, R.R., Zhang, H.Y., Wang, J.H., Lin, X.G. 2014. *Bacillus fengqiuensis* FJAT-14578 sp. nov., isolated from a typical sandy loam soil under long term NPK fertilization in the North China. *Inter. J. System. Evol. Microbiol*. 64, 2849-2856. doi: 10.1099/ijs.0.063081-0.

Supplementary Figure 4. Open reading frames (ORF) for koranimine synthesizing genes in *Bacillus* spp. The top arrangement of ORFs is for *Bacillus* sp. NK2003, for which proteomic evidence of koranimine production was found (Evans *et al.* 2011). *B. simplex* strains II3b11 and 30N-5 exhibit the same arrangement of ORFs as *Bacillus* sp. NK2003. The iturin genes of *B. subtilis* BG03 and the surfactin genes of *B. amyloliquefaciens* subsp. *plantarum* FZB43 line up with some of the ORFs of *B. simplex* and *Bacillus* sp. NK2003 as shown.

Evans, B.S., Ntai. I., Chen, Y., Robinson, S.J., Kelleher, N.L. (2011). Proteomics-based discovery of koranimine, a cyclic imine natural product. *J. Amer. Chem. Soc.* 133, 7316-7319.
