# Supplementary figures and images for: Mining the phytomicrobiome to understand how bacterial coinoculations enhance plant growth
Source: Front Plant Sci. 2015 Sep 24;6:784. doi: 10.3389/fpls.2015.00784 (PMC4585168; doi:10.3389/fpls.2015.00784)

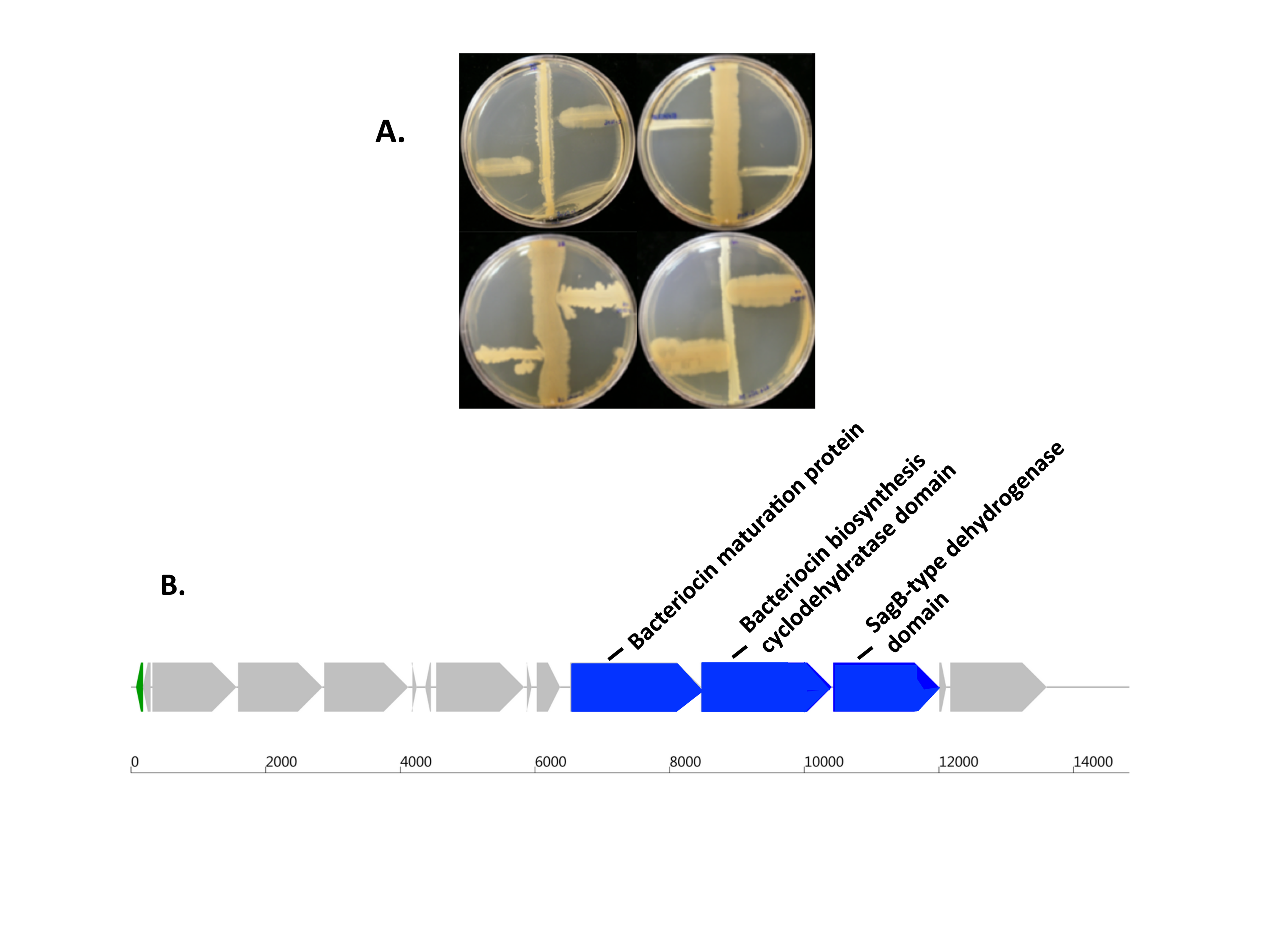

Supplement: Supplementary file 3 [file Image1.TIF]

# 95% family-wise confidence level

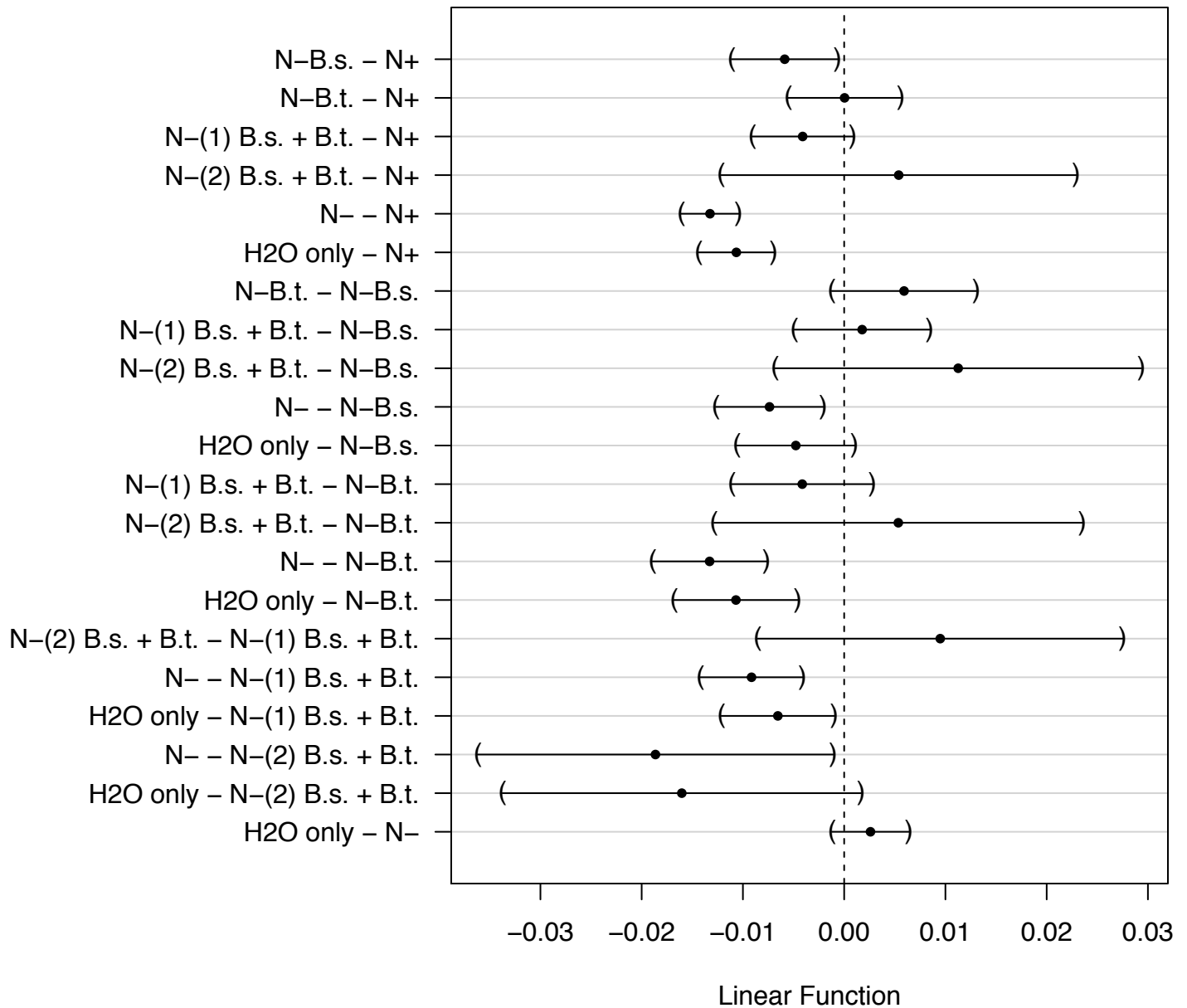

Supplement: Supplementary file 4 [file Image2.PDF]

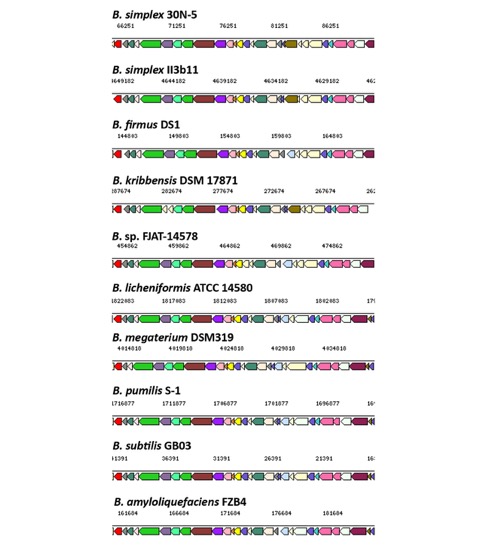

Supplement: Supplementary file 5 [file Image3.JPEG]

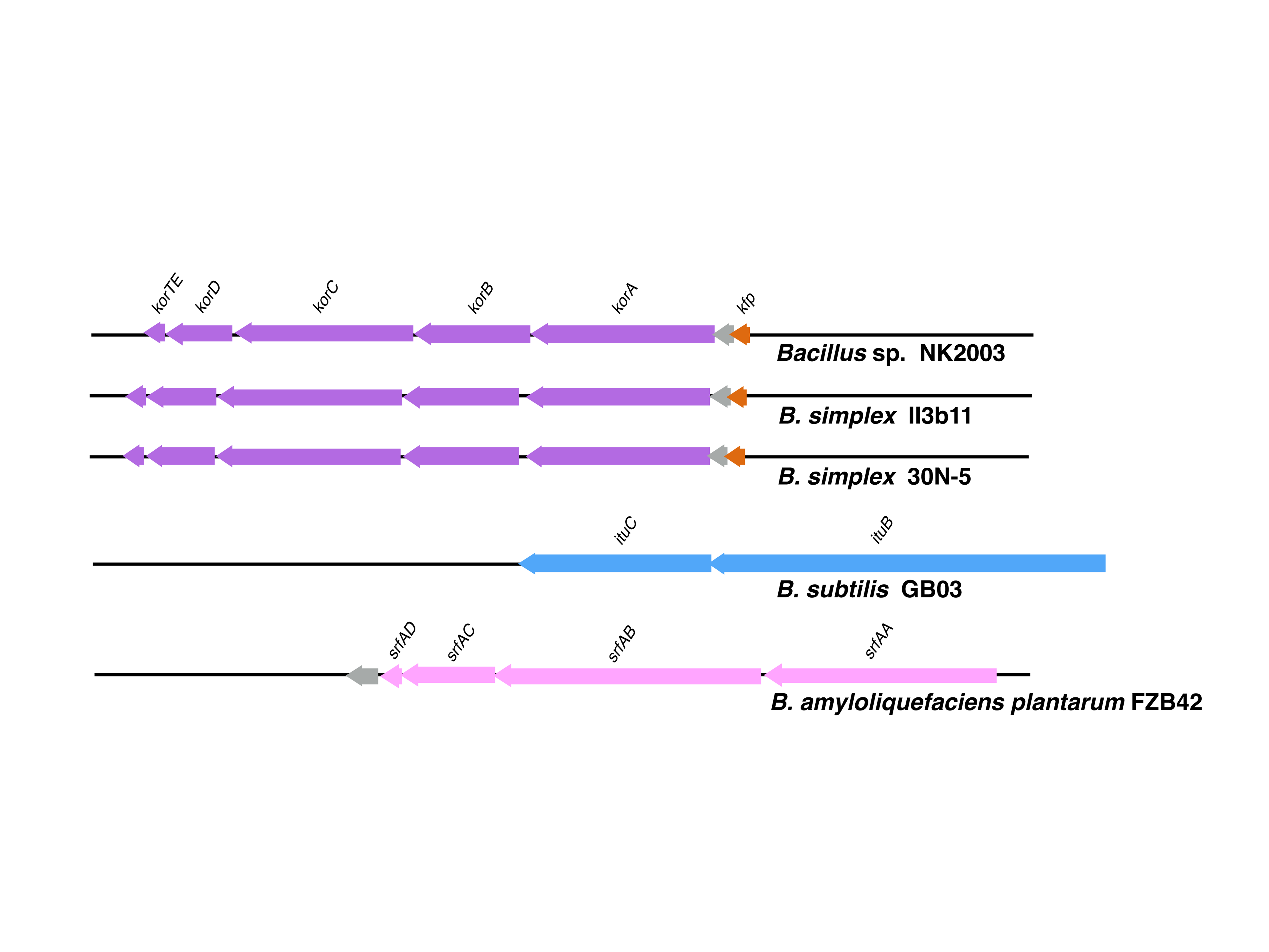

Supplement: Supplementary file 6 [file Image4.TIFF]
